# Investigating schoolwork engagement and mental health of children based on structural equation modeling

**DOI:** 10.1002/pcn5.70141

**Published:** 2025-07-01

**Authors:** Nobuko Egusa, Yoshiyuki Hirano, Takeshi Katayama, Eiji Shimizu

**Affiliations:** ^1^ The University of Osaka, United Graduate School of Child Development, The University of Osaka, Kanazawa University, Hamamatsu University School of Medicine, Chiba University, and University of Fukui, Suita Osaka Japan; ^2^ Katayama Pediatric Clinic Tsuyama Japan; ^3^ Research Center for Child Mental Development Chiba University Chiba Japan; ^4^ Department of Cognitive Behavioral Physiology Graduate School of Medicine Chiba Japan

**Keywords:** anxiety, depression, schoolwork engagement (SE), stress coping, structural equation modeling (SEM)

## Abstract

**Aim:**

This study explored whether depression, anxiety, social support, and coping with stress are related to schoolwork engagement (SE) using structural equation modeling.

**Methods:**

This study investigated 798 Japanese elementary and junior high school students (4th to 9th grades) aged 9–15 years (*M* = 13.9 years, *SD* = 1.79 years). This study used the Utrecht Work Engagement Scale for Students, Patient Health Questionnaire‐9 Items Modified for Adolescents, Generalized Anxiety Disorder‐7, Adolescent Coping Orientation for Problem Experiences, and Social Support for Children and Adolescents.

**Results:**

SE had no significant effect on anxiety or depression and vice versa. Coping with stress had a significant positive middle effect on SE (*β* = 0.509, *p* < 0.001) and a significant positive weak effect on anxiety (*β* = 0.225, *p* < 0.001). However, it did not have a significant effect on depression. Social support had a significant positive weak effect on SE (*β* = 0.175, *p* < 0.001). Moreover, it had a significant negative middle effect on anxiety (*β* = −0.378, *p* < 0.001) and a significant negative, weak effect on depression (*β* = −0.133, *p* < 0.01).

**Conclusion:**

Our study suggested that depression, anxiety, and SE have no relationship, and that strategies of coping with stress predict higher SE but also higher anxiety.

## Graphical abstract

Schoolwork engagement (SE) has no relationship with anxiety or depression. Although coping with stress might cause a slight increase in anxiety, it also contributes to fostering SE.

## INTRODUCTION

### Enhancing both academic success and mental health

In education, it is an important goal in society to ensure that children not only succeed academically, but also that they continue to grow and improve their mental health.^1^, ^2^ Schoolwork engagement (SE), which is said to be the holy grail of learning, has been emphasized in education.^3^ SE is defined as a persistent, positive, affective, and motivational state of fulfillment in children and adolescents associated with feelings of commitment to school.^4^ Researchers, educators, and policymakers are turning to SE to solve children's education and mental health problems.^2^ SE studies developed primarily in North America (North American approach) have been analyzed and appear to benefit school education outcomes. SE has additionally been seen as a means to address the problems of student boredom, alienation, low grades,^5^ school dropout,^6^ and problematic behaviors.^6^, ^7^ The main dimensions of SE within the North American approach include cognitive engagement, emotional engagement, behavioral engagement.^4^ On the other hand, another SE approach has been studied in recent years, mainly in Europe (European approach).^8^ The components of SE with a European approach focus on vigor, absorption, and dedication. The European approach is associated not only with academic success but also with indicators of well‐being (e.g., life satisfaction and career satisfaction).^9^, ^10^, ^11^ Some studies suggest that SE is also correlated with mental health.^12^, ^13^

### Specific contributors of SE and mental health

Currently, schools have been facing new challenges of digitalization, diversity, and mental health.^14^, ^15^ Children and adolescents spend more time in school.^16^ Though the school setting prevents depressive symptoms,^17^ SE may act as a buffer against these new risk factors.^18^, ^19^ To date, major motivational theories in the field of education exist, including self‐determination theory, which emphasizes competence, autonomy, and relatedness.^20^, ^21^ Another theory is expectancy theory, which relates the efforts and outcomes related to expectancy and instrumentality of performance.^9^, ^22^ It has been incorporated into schooling, focusing primarily on how individuals are motivated in terms of academic success. To confront new challenges, because study activities are goal‐oriented and evaluated like in the workplace, SE researchers using the European approach have focused on several similarities between studying and working.^11^, ^18^ Since researchers studied the replacement of work engagement (WE) with SE, they applied the job demands–resources (JD‐R) model to the workplace within the school context. WE is a positive, affective‐motivational state of fulfillment characterized by vigor, dedication, and absorption.^23^, ^24^ The JD‐R model clarifies how demands and resources interact and lead to well‐being at work. High job demands can lead to burnout and depressive symptoms that are related to psychological stress. High job resources, such as social support, and personal resources, such as optimism, self‐esteem, and stress‐coping skills, can lead to WE and good performance. Additionally, WE can reduce the symptoms of depression and anxiety.^25^, ^26^, ^27^

Previous SE studies developed study demand–resources (SD‐R) models.^11^, ^15^, ^18^, ^28^ These studies attempted to establish a clear framework for the assessment of student well‐being and its possible causes and consequences. Because existing educational theories alone are not sufficient to meet new educational needs, it is necessary to consider their relationship with and complement existing educational models based on scientific evidence.^28^

Depression and anxiety are the most common mental health problems experienced by children and adolescents. Meanwhile, a corresponding integration of evidence in the school resources and personal resources to enhance mental health via SE is lacking. Following the footsteps of the SD‐R model studies,^11^, ^15^, ^18^, ^28^ we attempt to apply the JD‐R model to well‐being at school to understand whether high school resources, such as social support, and high personal resources, such as stress‐coping skills, can lead to SE and reduce symptoms of depression and anxiety. Social support is an exchange of resources between at least two individuals perceived by the provider or recipient as intended to enhance the well‐being of the recipient.^17^, ^29^ Coping with stress is defined as constantly changing cognitive and behavioral efforts to manage specific external and/or internal demands appraised as taxing or exceeding personal resources.^30^

### Purpose

Our study aimed to (a) test the proposed hypotheses about the relationships linking SE, social support, and coping with stress, depression, and anxiety; and (b) evaluate the mental health of children and adolescents using a demands–resources model.

However, to the best of our knowledge, the relationship between mental health and SE as well as its contributors remain unclear. We explored these relationships using structural equation modeling (SEM).

### Hypotheses

**Figure 1:**
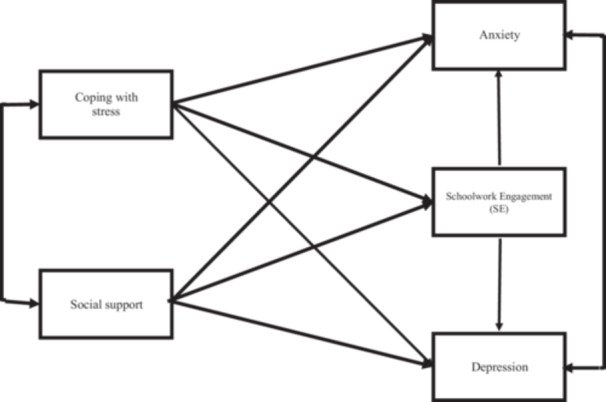
Hypothetical model of mental health of children and adolescents.

This study examined the following hypotheses: SE affects anxiety and depression (Hypothesis 1 [H1]); anxiety and depression affect SE, and vice versa (Hypothesis 2 [H2]); coping with stress affects SE (Hypothesis 3 [H3]); coping with stress affects anxiety (Hypothesis 4 [H4]); coping with stress affects depression (Hypothesis 5 [H5]); social support affects SE (Hypothesis 6 [H6]); social support affects anxiety (Hypothesis 7 [H7]); and social support affects depression (Hypothesis 8 [H8]). Figure 1 illustrates these hypotheses.

## METHODS

### Ethics approval

This study was approved by the Ethics Review Committee of Chiba University Graduate School of Medicine. The study was conducted in seven schools. With the cooperation of all relevant school principals, teachers, and physicians, written informed consent was obtained from the participating students and their parents.

### Participants

Data were collected from students attending four junior high schools (two public and two private schools) and three elementary schools (one public and two private schools) in the Chugoku and Kinki districts of Japan. They were recruited from the 4th to 9th grades and within the age group of 9–15 years.

In total, 853 students were assessed for eligibility. Thirty students without parental consent declined to participate in this study. Fourteen students who did not complete the questionnaire were excluded from the analysis. Subsequently, 798 students who received the assessment and responded to all questions with their parental consent and missing values were analyzed. Table S1 shows the missingness patterns. There were 610 junior high and 188 elementary school students.

### Measures

#### Utrecht work engagement scale for students (14‐question, 7‐point scale)

The Utrecht Work Engagement Scale for Students (UWES‐S) is initially made up of 17 subdivisions that measured adolescents’ feelings of commitment to school and scored on a 7‐point Likert scale ranging from 0 (*never*) to 6 (*always*).^31^ It has six subdivisions that evaluate vigor, five subdivisions that measure dedication, and six subdivisions that measure absorption. The scale reliability was found to have an *ω* of 0.81 and greatest lower bound (*glb*) of 0.85 for vigor, *ω* of 0.83 and *glb* of 0.84 for dedication, and *ω* of 0.84 and *glb* of 0.85 for absorption.^31^ Intercorrelations and internal consistencies (Cronbach's *α* on the diagonal) of the three subscales of the Japanese‐translated version of UWES‐S were sufficient, respectively.^32^

#### Patient health questionnaire‐9 Item modified for adolescents (9‐question, 4‐point scale)

The Patient Health Questionnaire‐9 Item Modified for Adolescents (PHQ‐A) was used to identify elevated depressive symptoms in adolescents.^33^ The original PHQ‐9 was modified to be developmentally appropriate for adolescents. Adolescents were asked to rate how frequently they had experienced symptoms in the past 2 weeks from 0 (*not at all*) to 3 (*nearly every day*).^34^, ^35^, ^36^, ^37^ The Japanese version has been developed^38^; however, in this study, we used eight questions without mentioning suicidal ideation (Item 9).

#### Generalized anxiety disorder‐7 (7‐question, 4‐point scale)

The Generalized Anxiety Disorder‐7 (GAD‐7) scale measures the frequency and severity of generalized anxiety disorder symptoms using seven items scored on a 4‐point Likert scale (0 = *not at all*, 1 = *on several days*, 2 = *half or more days*, and 3 = *almost daily*) in the previous 2 weeks.^39^ The Japanese version has been developed.^40^

#### Social support for children and adolescents (12‐question, 4‐point scale)

Social Support for Children and Adolescents (12‐question, 4‐point scale) asked participants, “Do your friends help you when you make mistakes?” with responses ranging from 1 (*never*) to 4 (*very suitable*). These questions aimed to measure perceived support. This refers to the expectation that support will be provided rather than referring to specific instances in which one has received support.^41^

#### Adolescent coping orientation for problem experiences (41‐question, 5‐point scale)

The Adolescent Coping Orientation for Problem Experiences (A‐COPE) scale was developed to measure adolescent coping behaviors. Analyses offer partial evidence for the internal reliability and concurrent validity of A‐COPE as an instrument for measuring adolescents’ coping with life problems.^42^ The adolescents were asked to record how often, on a five‐point Likert scale (i.e., *never*, *hardly ever*, *sometimes*, *often*, or *most of the time*) they used each behavior when they faced difficulties or felt tense. 6th is recreation and relaxation. The Japanese version has 41 questions on a 5‐point scale.^43^

### Statistical analysis

In addition, we use R (Version 4.2.3; The R Foundation for Statistical Computing) with the package “Lavaan” (Version 0.6‐16).^44^, ^45^ All packages (Amos, LISREL, EQS, and R) produce estimates that are comparable in accuracy.^46^ We chose to conduct a survey of statistics with R and HAD (Version 18‐002), a system for statistical programs with EXCEL.^47^ Multiple comparisons were performed using the Holm method and the MLR estimator was used to consider robustness for skewness. We examined multiple conformity indicators, such as the root‐mean‐square error of approximation (RMSEA), goodness‐of‐fit index (GFI), adjusted goodness‐of‐fit index (AGFI), and Tucker–Lewis index (TLI) to create a better model. As the surveys were conducted in multiple schools, we tested the homogeneity of variances. We checked the intraclass correlation coefficient (ICC) using HAD (Table 1). Because the ICC level was poor, we conducted a multilevel modeling approach using R. Multilevel SEM and SEM methods were compared using the chi‐square difference test; however, significant differences indicated that the model with multilevel SEM methods should be rejected.^48^

**Table 1:** Intraclass correlation coefficient, Variance, and reliability on the study variables.

Variables	Efficient (*N* = 798)	ICC	95% CI Lower	95% CI Upper	DE	Reliability	Df 1	Df 2	*F*‐value	*p*‐value
SE		0.147	0.058	0.452	16.684	0.949	6	791	19.555	0.000
Support		0.028	0.006	0.143	3.992	0.757	6	791	4.107	0.000
Coping with stress		0.130	0.050	0.418	14.848	0.941	6	791	17.060	0.000
Anxiety		0.024	0.004	0.128	3.577	0.727	6	791	3.665	0.001
Depression		0.028	0.006	0.141	3.939	0.753	6	791	4.050	0.001

*Note*: Column 2 represents the samples excluding the missing values of the data.

Abbreviations: CI, confidence interval; DE, variance; ICC, intraclass correlation coefficient; SE, school engagement.

## RESULTS

### Demographics

The demographic characteristics of the participants are shown in Table 2. The mean age of the participants was 13.9 years, *SD* = 1.79 years, and the range was 9–15 years. There were more females (62.9%) than males (38.1%). The proportion of junior high school students (76.4%) was higher than elementary school students (23.6%). The variables, means, standard deviations, minima, maxima and Cronbach's α are listed in Table 3 showed SE (*Mean* = 2.8, *SD* = 1.3), social support (*Mean* = 3.2, *SD* = 0.7), coping with stress (*Mean* = 3.1, *SD* = 0.7), anxiety (*Mean* = 0.8, *SD* = 0.7), and depression (*Mean* = 0.9, *SD* = 0.7). The alpha levels for various measures indicated an acceptable level of inter‐item consistency.

**Table 2:** Descriptive demographic statistics of study participants (*n* = 798).

Variables	Mean	SD	Range
Female	497 (62.9%)		
Male	301 (38.1%)		
Junior high school	610 (76.4%)		
Elementary school	188 (23.6%)		
Age (years)	13.9	1.79	9.0–15.0

**Table 3:** Summary statistics of the dataset including mean, standard deviation minimum, maximum, and Cronbach's α for this study.

Efficient (*N* = 798)					
Variables	Mean	SD	Minimum	Maximum	Cronbach's α
*SE*	2.8	1.3	0.0	6.0	0.950
*Social support*	3.2	0.7	0.0	4.0	0.930
*Coping with stress*	3.1	0.7	0.0	4.9	0.936
*Anxiety*	0.8	0.7	0.0	3.0	0.856
*Depression*	0.9	0.7	0.0	2.9	0.842

*Note*: (Effcient *N* = 798) represents sample numbers excluding the missing values of the data.

Abbreviation: SE, school engagement.

### Correlation analysis

Table 4 shows the correlations among the five variables. This indicates that virtually all correlations were in the expected direction. Multiple comparisons are performed to clarify the effect sizes (Δχ^2^(*df* = 4) = 2017.992, *p* < 0*.001, η* ^*2*^ = 0.506; *95% CI* = 0.463).

**Table 4:** Correlation analysis.

Variables	SE	Social support	Stress coping	Anxiety	Depression
*SE*	1.000				
*Social support*	0.510**	1.000			
*Stress coping*	0.625**	0.655**	1.000		
*Anxiety*	−0.100**	−0.227**	−0.005	1.000	
*Depression*	−0.069	−0.131**	−0.038	0.399**	1.000

**p < .01.

Abbreviation: SE, school engagement.

### Fit of models

**Figure 2:**
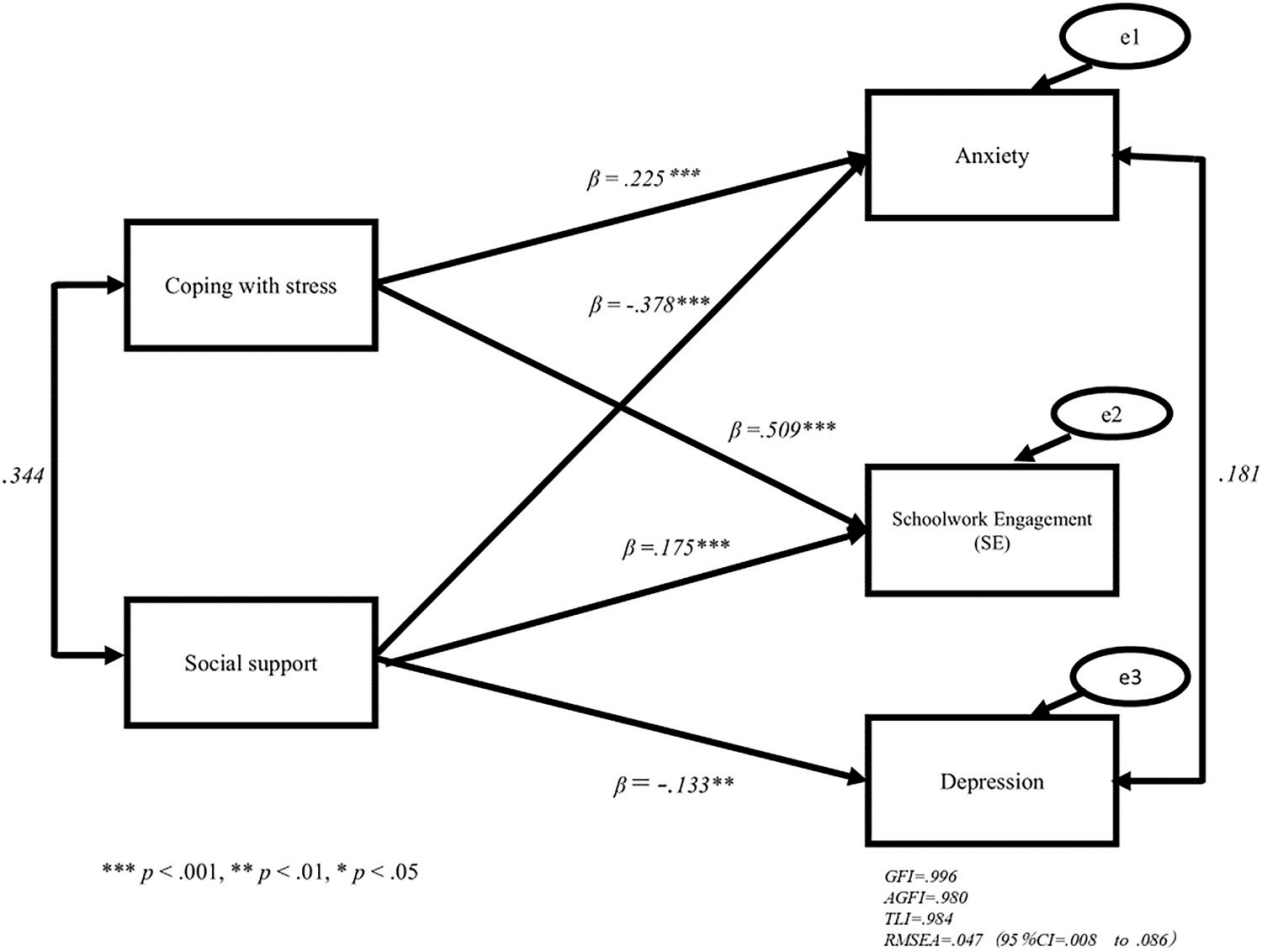
Psychological model for children and adolescents of mental health Model 1. ***p < 0.001, **p < 0.01, *p < 0.05. goodness‐of‐fit index (GFI) = 0.996, adjusted goodness‐of‐fit index (AGFI) = 0.980, Tucker–Lewis index (TLI) = 0.984, root‐mean‐square error of approximation (RMSEA) = 0.047 (95% CI = 0.008 to 0.086).

Before comparing the competing models and examining the hypothesized relationships in the structural equation analysis, the measurement models were tested using confirmatory factor analysis (Table 5, Table S2). Table 6 shows the results (Model 3 (Δχ2 (*df* = 3) = 2591.017, *p* < 0.001), with RMSEA = 1.040, GFI = 0.799, AGFI = −0.340, and TLI = −7.045). Although Model 3 showed a fit to the data, some fit indices were unacceptable. Model 2 showed a fit to the data, with some of the fit indices meeting their respective criteria for an acceptable fit (Model 2 (Δχ2 (*df* = 0) = 0.000, *p* < 0.001), with RMSEA = 0.000, GFI = 1.000, AGFI = 1.000, and TLI = 1.000). Model 2 was satisfactory and some parameter estimates were not significant (*p* > 0.05). Figure S1, Table 7, Figure 2 and Table 8 show the results (Model 1 (Δχ2 (*df* = 3) = 8.190, *p* < 0.001), with RMSEA = 0.047, GFI = 0.996, AGFI = 0.980, and TLI = 0.984). Model 1 was satisfactory and all parameter estimates were significant (*p* < 0.01). Therefore, we adopted this model. We examined the possible reverse effects and vice versa. Table S3 shows **Model R3** (Δχ2 (*df* = 3) = 2575.197, *p* < 0.001, with RMSEA = 1.037, GFI = 0.807, AGFI = −0.285, and TLI = − 6.995). Figure S2 and Table S4 show **Model R2** (Δχ2 (*df* = 0) = 0.000, *p* < 0.001, with RMSEA = 0.000, GFI = 0.000, AGFI = 1.000, and TLI = 1.000).

**Table 5:** Fit of models that specify the relationship between variables (*N* = 798).

Models	*χ*²	*df*	*RMSEA*	CI lower	CI upper	*GFI*	*AGFI*	*TLI*
*Model 1*	8.190	3	0.047	0.008	0.086	0.996	0.980	0.984
*Model 2*	0.000	0	0.000	0.000	0.000	1.000	1.000	1.000
*Model 3*	2591.017	3	1.040	1.006	1.074	0.799	−0.340	−7.045
*Modified M1*	94.693	5	.150	0.124	0.177	0.957	0.872	0.833

Abbreviations: AGFI, adjusted goodness‐of‐fit index; CI, confidence interval; GFI, goodness‐of‐fit index; RMSEA, root‐mean‐square error of approximation; TLI, Tucker–Lewis index.

**Table 6:** Model 3 standardized on the study variables (*β*‐values).

	Estimate	*p* value	Lower	Upper
*Support → Depression*	−0.748	0.000	−1.140	−0.357
*Coping → Depression*	0.455	0.090	−0.071	0.981
*Support → Anxiety*	−1.514	0.000	−1.840	−1.188
*Coping → Anxiety*	1.245	0.000	0.641	1.850
*Support* → *SE*	0.153	0.035	0.011	0.295
*Coping* → *SE*	0.788	0.000	0.647	0.929
*SE → Depression*	−0.090	0.413	−0.306	0.126
*SE → Anxiety*	−0.197	0.074	−0.413	−0.019

*Note*: Adjusted goodness‐of‐fit index = −0.340; goodness‐of‐fit index = 0.799; root‐mean‐square error of approximation = 1.040 (95％ confidence interval = 1.006 to 1.074); Tucker–Lewis index = −7.045.

Abbreviation: SE, school engagement.

**Table 7:** Model 2 standardized on the study variables (*β*‐values).

	Estimate	*p* value	Lower	Upper
*Support → Depression*	−0.182	0.001	−0.287	−0.076
*Coping → Depression*	0.107	0.073	−0.010	0.225
*Support → Anxiety*	−0.380	0.000	−0.473	−0.287
*Coping → Anxiety*	0.304	0.000	0.186	0.423
*Support* → *SE*	0.175	0.000	0.096	0.255
*Coping* → *SE*	*0.509*	0.000	0.436	0.581
*SE → Depression*	−0.042	0.382	−0.137	0.052
*SE → Anxiety*	−0.095	0.053	−0.192	0.001

*Note*: Adjusted goodness‐of‐fit index = 1.000; goodness‐of‐fit index = 1.000; root‐mean‐square error of approximation = 0.000 (95％ confidence interval = 0.000 to 0.000); Tucker–Lewis index = 1.000.

Abbreviation: SE, school engagement.

**Table 8:** Model 1 standardized on the study variables (*β*‐values).

	Estimate	*p* value	Lower	Upper
*Support → Depression*	−0.133	0.002	−0.215	−0.051
*Support → Anxiety*	−0.378	0.000	−0.468	−0.288
*Coping → Anxiety*	0.225	0.000	0.135	0.315
*Support* → *SE*	0.175	0.000	0.099	0.252
*Coping* → *SE*	0.509	0.000	0.436	0.581

*Note*: Adjusted goodness‐of‐fit index = 0.980; goodness‐of‐fit index = 0.996; root‐mean‐square error of approximation = 0.047 (95％ confidence interval = 0.008 to 0.086); Tucker–Lewis index = 0.984.

Abbreviation: SE, school engagement.

We attempted to clarify the other models with indirect effects (Model S1). An inspection of the modification indices for the models revealed that the model fit could not be improved within the allowed limits. Figure S3 and Table S5 showed **Modified Model 1** (Δχ2 (*df* = 5) = 94.693, *p* < 0.001), with RMSEA = 0.150, GFI = 0.957, AGFI = 0.872, and TLI = 0.833; and Figure S4 and Table S6 showed **Modified Model 2** (Δχ2 (*df* = 3) = 63.737, *p* < 0.001), with RMSEA = 0.159, GFI = 0.970, AGFI = 0.851, and TLI = 0.811).

### SEM results

The standardized path coefficients are shown in Table 8. SE has no significant effect on anxiety or depression. Coping with stress had a significant positive middle effect on SE (*β* = 0.509, *p* < 0.001). Coping with stress had a significant positive, weak effect on anxiety (*β* = 0.225, *p* < 0.001). Social support had a significant positive, weak effect on SE (*β* = 0.175, *p* < 0.001), a significant negative middle effect on anxiety (*β* = −0.378, *p* < 0.001), and a significant negative, but weak, effect on depression (*β* = −0.133, *p* < 0.001) (Figure 2). Neither anxiety nor depression had a significant effect on SE.

## DISCUSSION

As no previous studies have explored the causal relationship between SE, depression, and anxiety, this study aimed to test the validity of our hypotheses on the relationship between anxiety, depression, coping with stress, social support, and SE. Contrary to the findings from prior studies on how WE can reduce symptoms of depression and anxiety in adults, we found that SE did not have any significant impact on symptoms of depression and anxiety in children and adolescents, and vice versa (H1 and H2 were not confirmed). In this respect, our results contradict our hypotheses. However, social support and coping with stress were found to increase SE (H3 and H6 were confirmed), which is consistent with the results of previous studies on children and adolescents.

A previous study found that SE mediates symptoms of psychological distress and academic achievement after peer victimization.^49^ Thus, SE is correlated with mental health.^12^, ^13^ Regarding WE, two studies revealed that WE was related to or predicted depressive symptoms.^25^, ^26^ While one study showed that depressive symptoms predicted WE and vice versa,^50^ another study showed that depressive symptoms and anxiety predicted WE and vice versa.^27^ Although WE and SE are the same concepts, SE is characterized by three distinct perspectives (psychological, educational, and developmental^16^) and four unique contexts (students, peers, classroom, and school environment).^16^, ^51^ These differences may have affected our research. Coping with stress had a significant positive and weak effect on anxiety (H4) and had no significant effect on depression (H5). They are not in accordance with the correlation analysis.^52^ Some previous studies on negative coping^53^ and avoidant coping^54^ in relationship to a decrease in mental health are consistent with our findings. Social support had a significant negative intermediate effect on anxiety and a significant negative weak effect on depression, confirming H7 and H8. Previous studies on how social support decreases somatic symptoms^54^ and depressive symptoms^55^, ^56^ are consistent with our findings. Our findings are also consistent with those of previous studies on problem‐focused coping^57^; adaptive coping, problem‐solving and help‐seeking^58^; social‐emotional skills increasing SE^59^; and how peers, classmates, teachers, and parents can increase SE.^60^ Recently, research on SD‐R models and SD‐R theories has been progressing rapidly, but has not yet been finalized.^11^, ^28^, ^61^, ^62^ If the intention is to respond to new risks and improve children's mental health as well as academic success through education, a new integrated theoretical framework via SE would be needed to shape child and adolescent learning. Further studies are required to clarify the relationships among SE, anxiety, depression, and resources.

### Limitations

This study has the following limitations. Our findings on the relationship between anxiety, depression, coping with stress, social support, and SE could not be confirmed as this was a cross‐sectional study, and longitudinal research is needed to discuss the causal relationships among these factors.^63^ In addition, we used a non‐probability sampling method to collect data. Future studies should use random sampling.^64^

### Conclusion

In conclusion, our study suggested two findings: (1) depression, anxiety and SE have no relationship; and (2) strategies of coping with stress predict higher SE but also higher anxiety.

## AUTHOR CONTRIBUTIONS

Nobuko Egusa and Eiji Shimizu developed the conception and design of the study. Nobuko Egusa and Takeshi Katayama acquired data. Nobuko Egusa and Yoshiyuki Hirano analyzed the data. Nobuko Egusa drafted the manuscript and figures. All of them read and checked the paper.

## CONFLICT OF INTEREST STATEMENT

The authors declare no conflicts of interest.

## ETHICS APPROVAL STATEMENT

This study was conducted after approval by the ethics review committee of the Chiba University Graduate School of Medicine.

## PATIENT CONSENT STATEMENT

With the cooperation of school principals, teachers, and a physician who was the director of a pediatric clinic, informed assent from the children and informed consent from their parents in writing were obtained for the study.

## CLINICAL TRIAL REGISTRATION

We received no clinical trial registration since this was not an interventional study.

## Supporting information

CSC SupplementaryTables 20250424.

CSC SupplementaryFigures 20250507.

## Data Availability

We did not obtain consent from the participants to share the data, so supporting data is not available.
