# Supplementary figures and images for: Investigating schoolwork engagement and mental health of children based on structural equation modeling
Source: PCN Rep. 2025 Jul 1;4(3):e70141. doi: 10.1002/pcn5.70141 (PMC12213602; doi:10.1002/pcn5.70141)

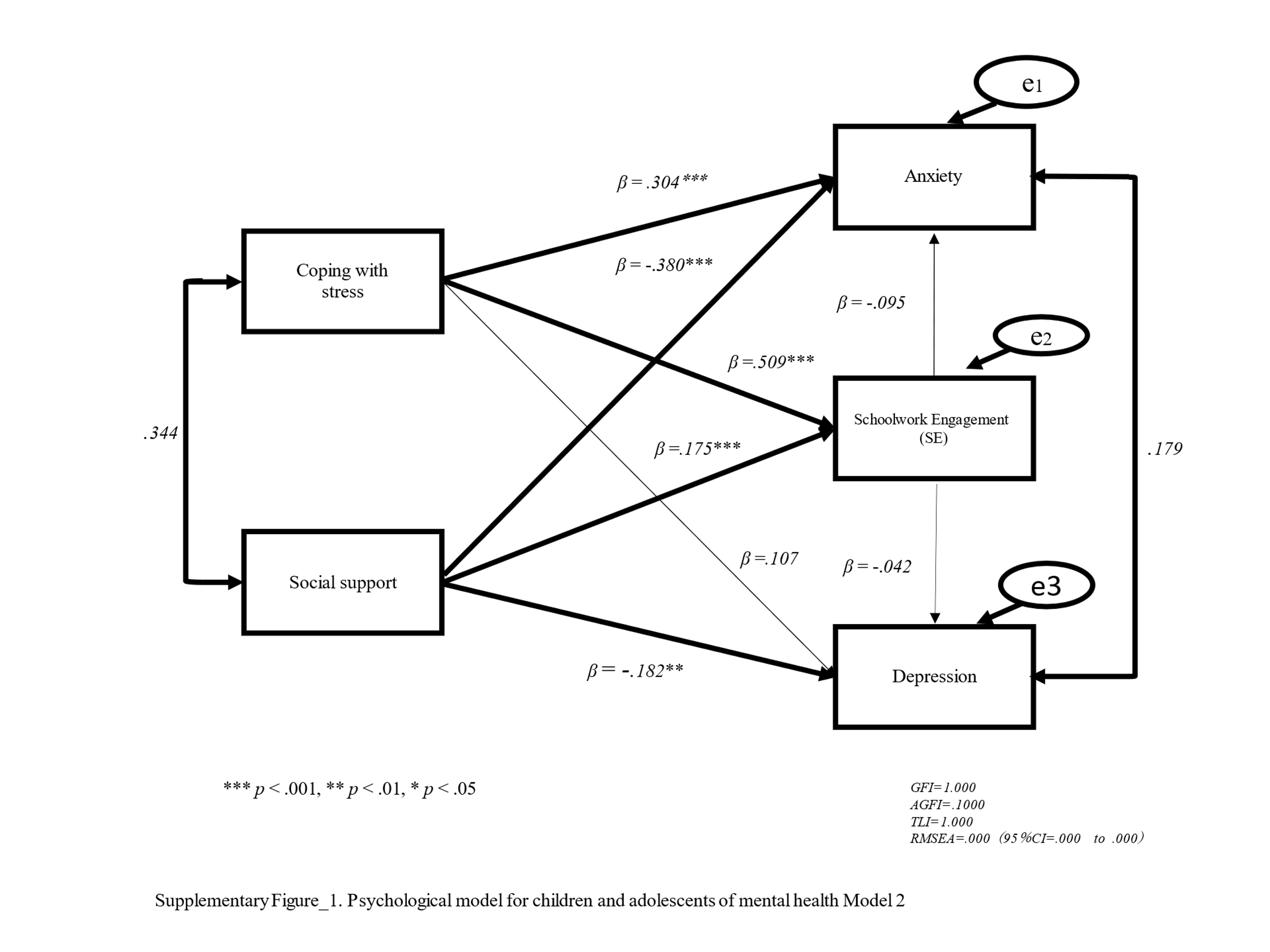


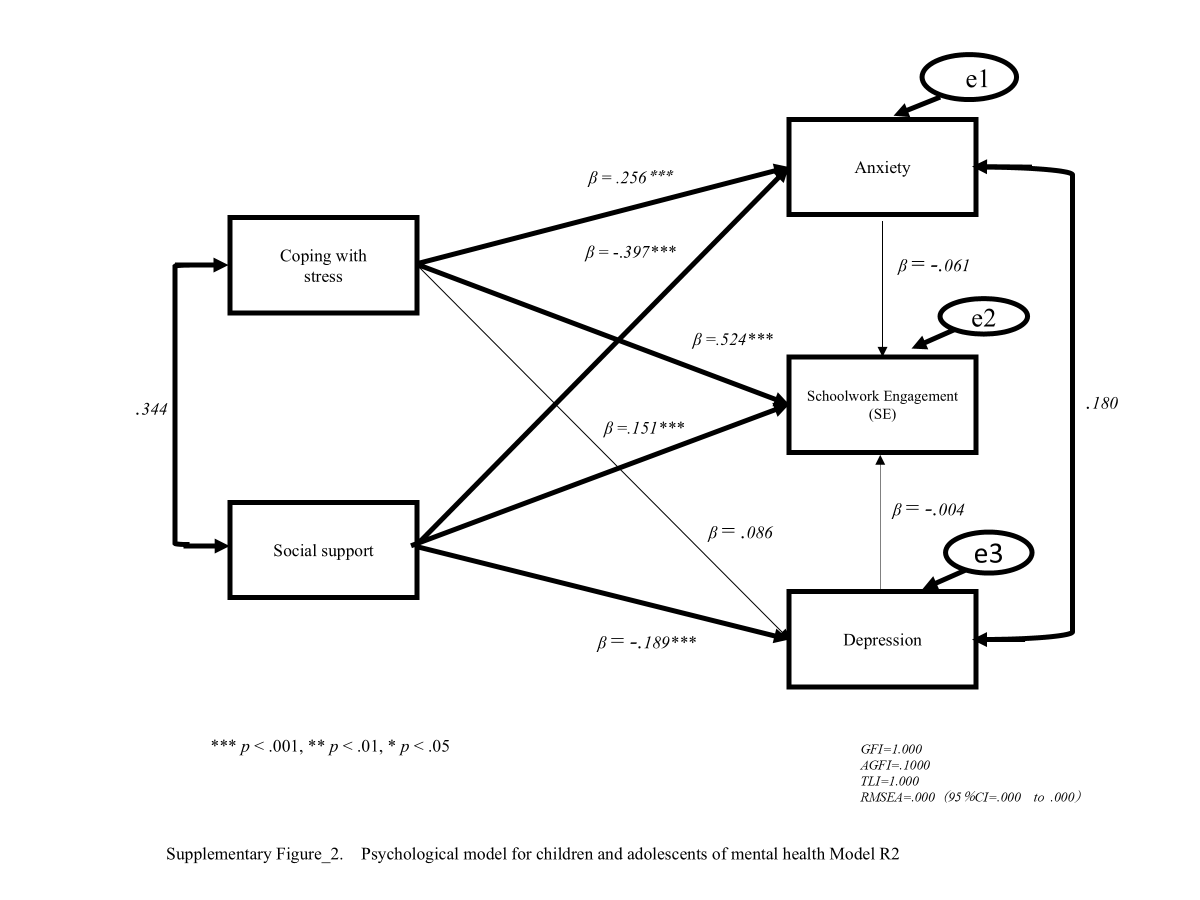


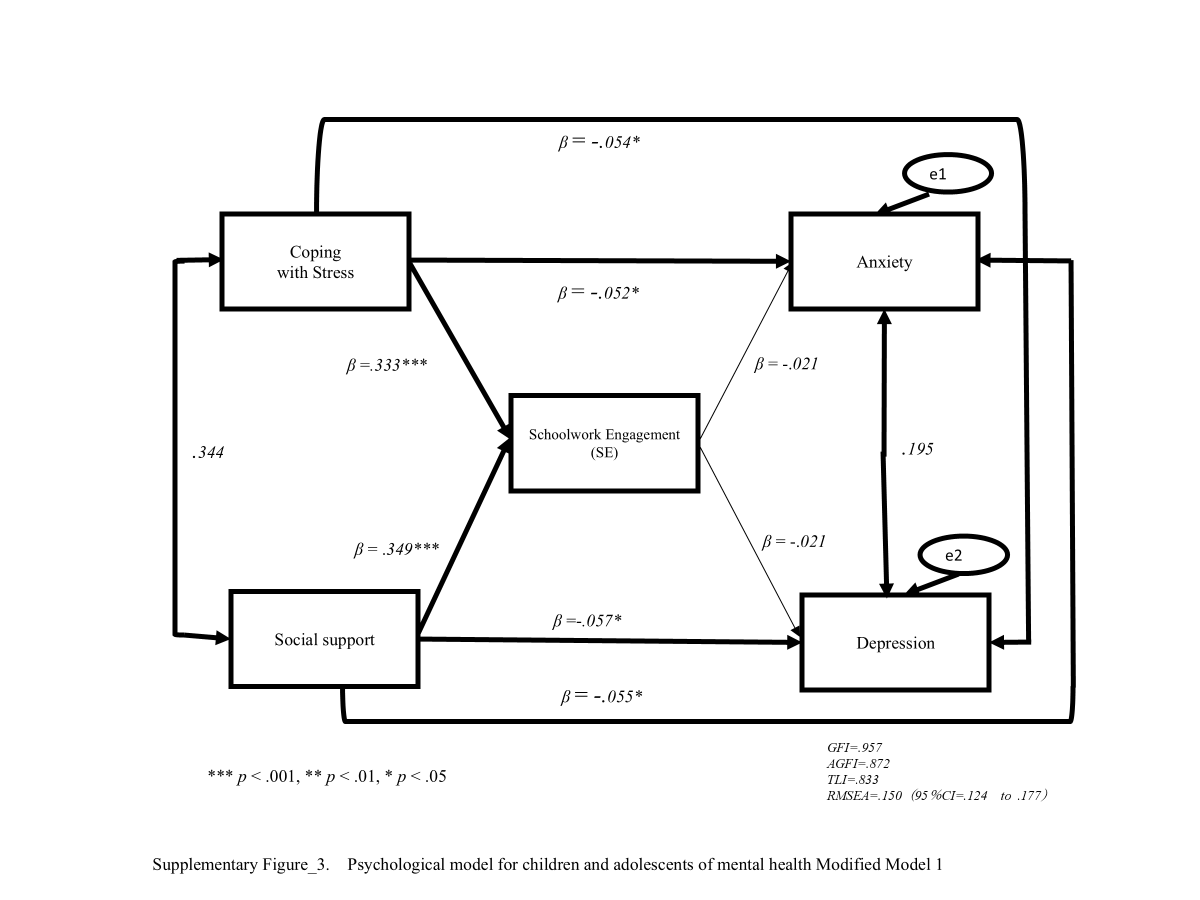


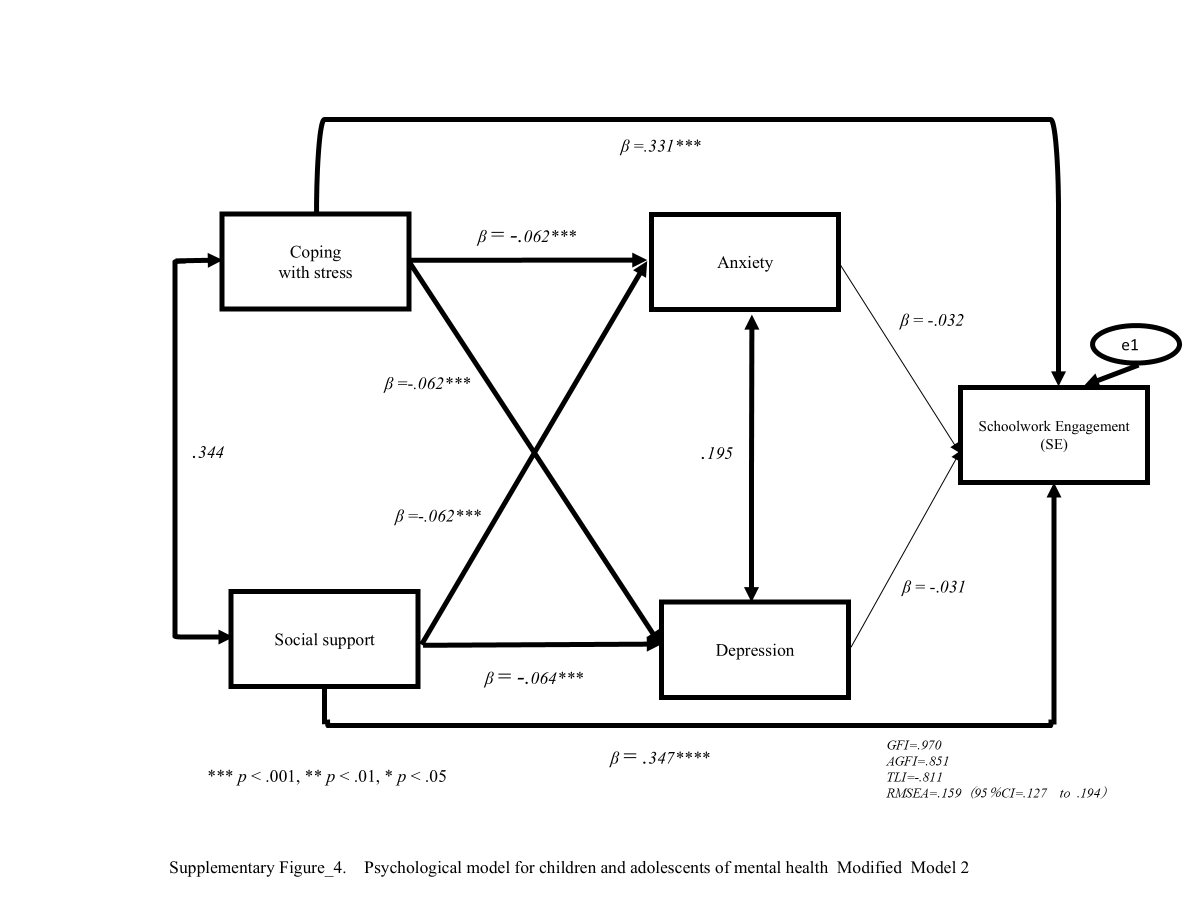

Supplement: Supplementary file 2 — CSC SupplementaryFigures 20250507. [file PCN5-4-e70141-s001.docx]
