# Supplementary material for: Investigating schoolwork engagement and mental health of children based on structural equation modeling
Source: PCN Rep. 2025 Jul 1;4(3):e70141. doi: 10.1002/pcn5.70141 (PMC12213602; doi:10.1002/pcn5.70141)
Supplement: Supplementary file 2 [file PCN5-4-e70141-s002.docx]

Supplementary Table1　The display of patterns of missinngness

Number of participants (*n* =809)	*SE*	*Social support*	*Coping with stress*	*Deppression*	*Anxiety*	*Total missing*
57		x				
230				x		
232					x	
233					x	
409		x				
449				x		
582				x		
589				x		
609		x			x	
687						
694				x		
Total missing	0	4	0	5	3	11

Supplementary Table2 Fit of reversed cuasal Models that Specify the Relationship between Variables (N = 798)

Models	χ^2	*df*	*RMSEA*	*ci.lower*	*ci.upper*	*GFI*	*AGFI*	*TLI*
Model1	8.190	3	.047	.008	.086	.996	.980	.984
ModelR2	.000	0	.000	.000	.000	1.000	1.000	1.000
ModelR3	2575.197	3	1.037	1.003	1.070	0.807	-0.285	-6.995
Modified M2	63.737	3	.159	.127	.194	.970	.851	.811

Suppprementary Table3 Model R3 Standardized on the Study Variables (β-valu

	Estimate	*P value*	*lower*	*upper*
Support→ *Depression*	-.768	.000	-1.139	-0.397
Coping → *Depression*	.359	.108	-0.078	.796
Support → *Anxiety*	-1.523	.000	-1.849	-1.197
Coping → *Anxiety*	1.008	.000	.521	1.496
Support → *SE*	.098	.141	.033	.229
Coping→ *SE*	.823	.000	.693	.952
Depression → *SE*	-.017	.187	-0.043	.008
Anxiety→ *SE*	- .046	.002	-0.076	-0.016

GFI =.807
AGFI=-.285
TLI=- 6.995
RMSE=1.037 (*95%CI=1.003 to 1 .070*)

Suppromentary Table4 ModelR2 Standardized on the Study Variables (β-values)

	Estimate	*P value*	*lower*	*upper*
Support→ Depression	-.189	.000	-.289	-.089
Coping → Depression	.086	.094	-.014	.186
Support → Anxiety	-.397	.000	-.489	-.305
Coping → Anxiety	.256	.000	.153	.358
Support → SE	.151	.000	.071	.023
Coping→ SE	*.524*	.000	.448	.601
Depression → SE	-.004	.905	-.062	.055
Anxiety→ SE	- .061	.061	-.124	.003

GFI=1.000

AGFI=1.000

TLI=1.000

RMSEA=.000 (95%*CI=.000 to .000*)

Supplementay Table5 Modified Model 1 Standardized on the Study Variables (β-values)

	Estimate		P value	lower	upper
	direct	indirect			
Support→ *Depressioı*	-.057		.045	-.113	-.022
Coping → *Depressioı*	-.054		.043	-.102	-.021
Support → *Anxiety*	-.055		.044	-.103	-.021
Coping → *Anxiety*	-.052		.042	-.098	-.020
Support → *SE*		.349	.000	.327	.369
Coping → *SE*		.333	.000	.308	.356
SE→ *Depression*		-.021	.614	-.045	.026
SE→ *Anxiety*		-.021	.616	-.047	.027

GFI=.957

AGFI=.872

TLI=.833

RMSEA=.150 （*95%CI=.124 to .177*）

Supplemntary Table6 Modified Model 2 Standardized on the Study Variables (β-values)

Estimate			*P value*	*lower*	*upper*
	direct	*indirect*			
Support→ Depression		-.064	.003	-.107	-.022
Coping → Depression		-.062	.003	-.102	-.021
Support → Anxiety		-.062	.003	-.103	-.021
Coping → Anxiety		-.059	.003	-.098	-.020
Support → SE	.348		.000	.327	.369
Coping → SE	.332		.000	.308	.356
Depression → SE		-.009	.605	-.045	.026
Anxiety→ SE		-.010	.605	-.047	.027

GFI=.970
AGFI=.851
TLI=.811
RMSEA=.159 (*95%CI=.127 to .194*)
